# Hospital Planning in America

**Published:** 1922-03

**Authors:** 


### HOSPITAL PLANNING IN AMERICA.

AT a moment when England is specially interested in American Architecture, the new edition of Mr. Edward F. Stevens's "American Hospital of the Twentieth Century"* makes an opportune appearance. The author has added over 150 illustrations to the work, and introduced some fresh material. It is true that a very large number of the plans are still printed without any attached scale-indication or with scales so reduced in printing that the figures on them are illegible. We note this little blemish more as a matter of conscience on our part than as an actual bar to the usefulness of the book, for an expert user of the work can generally set up his own scale by deduction from the size of a bed, or from a staircase, or some other feature of the plan which is of standard size. But why not give the reader a help in this respect ?

The moment an Englishman falls upon any illustrations by American architecture he becomes aware of two things which fever his imagination and double his despair. We all know that the States are a land flowing with milk and honey, but this opulence is brought home to us with special vigour whenever we study their architectural opportunities. No Einstein could abolish the conditions of space more completely than do the Americans, and as for money, it seems to multiply over there like rabbits, or, to use a more appropriate comparison, like the number of German marks to a pound sterling. Seriously speaking, one does indeed seem to observe that conditions of site restriction are in these hospital schemes almost non-existent, and unless the badness of some of the elevations is due to parsimony, funds would appear to be virtually unlimited. This latter symptom is due, we have no doubt whatever, to the public spirit of the country, and is a wholly commendable sign, for to do him justice the American Hospital Architect does not starve his interior work to feed his elevations.

"The hospital," says Mr. Stevens, "which would be perfect and which would fulfil the climatic conditions of Southern France, however, would be entirely unsuitable in our northern States." How true this is ! But it does not prepare us for the discovery that throughout the States, and apparently in Canada also, the problem set before the designer is so wholly different from that which confronts the European architect. The American's task is not, it seems, harder but infinitely easier than ours.

What would not an English architect say in words of fervent thanksgiving if he were told when entering a hospital competition that he might follow these four remarkable American axioms :—

1. Cross ventilation (by windows) in large wards is not indispensable.
2. Internal corridors can be practically unlighted by windows.
3. No isolation or "cut-offs" are necessary or desirable for sanitary groups, nor are separate (or any) windows necessary for w.c.'s.
4. Tubular or flue ventilation can be used throughout.

With these permissions the plan problem almost disappears. We are far from wishing to assert that climatic conditions do not authorise and demand entirely fresh solutions in America of the problems which beset us here. But there are sundry fundamental facts of human nature, which it seems to us must be somewhat outraged by the American disregard of some of our cherished ideas on the separation of tainted air from air to be breathed by patients and their patient attendants. Take, for example, the plan on page 176 of this book which illustrates the Haynes Memorial Hospital at Brighton, Mass., in which Mr. Stevens had a hand, probably as consultant. In the typical pavilion there are wards at each end, one of ten, the other of eight beds. Good wards these, and like English wards save for the American absence of sanitary blocks attached thereto, and for the fact that, scaling from the beds in the absence of a printed scale, the floor allowance is very greatly under 100 square feet per bed. Between these two wards runs the centre block of the pavilion, which contains five private wards, one (two-bed) ward, a small operating sink, a ward pantry, a dining-room, five w.c.'s (all without direct windows), and a very crowded utility room. All these compartments open into a central corridor which we calculate to be some 7 ft. 6 in. wide, and 80 ft. long. It is entirely windowless, and though no doubt it derives borrowed light from the ward doors at each end, perhaps also from all the other doors, and from the main staircase (itself ill-lighted according to our ideas), it appears from the plan to be, apart from mechanical ventilation, a dark and contaminated air-tube, destined to relieve its own distress by puffing into one or other of the end wards according to the wind pressure available.

Of course the answer to these apparently insoluble mysteries is, that daylight is better in America than here, that artificial light is not objected to, and that mechanical ventilation does the rest. But when all is said it remains a fact that these American plans are not for our learning. Mechanical ventilation is not on the up-grade in this country, and it is most unlikely that the advantages of fresh air and, in some degree, isolation of sanitary accommodation will ever be completely thrown overboard by our hospital authorities. The example just quoted was taken at random from the first page at which the book opened, but instances can be multiplied, for the system is the normal one employed.

We confess to having had a hope that Mr. Stevens would give in his new edition some more extended explanation of the mechanical ventilation upon which apparently the habitability of these scantily windowed blocks of buildings must depend. But we find very little additional comfort on this, to us, important problem. Only four pages are devoted to it in a general chapter on heating and plumbing, and these pages—beyond a very insufficient discussion on the relative merits of plenum and exhaust systems, and a

* "The American Hospital of the Twentieth Century." By Edward P. Stevens, architect. Revised Edition, 1921. (New York: The Architectural Record Co. $7.50.)

modified expression of preference for the latter—contain no genuine explanation of the methods adopted. Probably the reason for this is that mechanical flue-ventilation being such a commonplace feature of American architecture, it appears unnecessary to describe it; but it seems difficult for us Britons to work ourselves into the condition of believing that a network of flues, however often cleaned, can really be satisfactory adjuncts to a building dedicated to man's battle with the germ-enemy of mankind.

The index of the work is not wholly satisfactory. We do not wish to be unduly critical about such a point, but must confess that when the book is again revised it would be well to see that the index of illustrations is either combined with the general index or clearly headed as such on every page. We interpolate this remark here, as we have had some difficulty in using the index as a guide to the essential information which, owing to the rather unsystematic nature of the literary arrangement, is often concealed in unlikely places.

Mr. Stevens's doctrine as to square and cubic space per bed is to be found in a single sentence on page 28. He is satisfied with 1,000 cubic feet per patient, looks on 12 feet as the maximum ceiling height, tolerates 10 feet (and would, on grounds of *appearance*, not permit less). He would like 100 square feet floor area per patient, but will consent to 83, this being, of course, the figure which approximately gives 1,000 cubic feet with a 12-foot ceiling. Opinions differ largely upon this topic among English experts, and we are far from wishing to dogmatise about it. In any case, as the American practice is probably scientifically based on the ascertained rate at which the atmosphere of the wards is mechanically changed; no rules of ours would be fair criteria for American conditions.

English readers of this work will be struck by the prevalence in the American continent of the single ward system. A very clear illustration of this is given in the plans (pp. 44-45) of the private pavilions of the Victoria General Hospital at Halifax, N.S. The second-floor plan, we take it, is a typical one; it contains eighteen one-bed wards, each about 16×10, one two-bed ward 16×14, and two three-bed wards. Every room has its own wash-hand basin, there are two w.c.'s available for 24 of the 26 patients on the floor, and one bath-room (windowless), also for common use. Two of the single rooms are evidently luxury rooms; they share a balcony between them, and share also a bath-room (with w.c. in it), which is windowless, and stands between the two rooms, with a door opening into each. The centre corridor is 150 feet long; it is lighted by a door on to a balcony at each end, and in the centre by a similar balcony door at the end of a transverse corridor. The staircase from which other light might come is shut off by a glazed screen. On this plan the vertical ventilation and heating ducts are clearly indicated. The remaining apartments on this floor are a sewery 17×13, a sink-room 12×10, a linen room, and of course the lift well.

It is difficult for a British writer to set down in words this description of a typical ward floor without seeming to be adversely criticising the scheme of the design. We do not wish to appear in such a light. It would be unfair to suggest that any of the details above enumerated are bad from the transatlantic point of view; even that bath and "toilet," stuffed windowless between two bedrooms and opening into both of them, may, for all we know, be a means of prolonging life; but the moral of the quoted plan, and, indeed, the moral of the whole of this exposition of American hospital design, is that we dare not even begin to try to learn from it. Why not? Because for our country we have certain standard requirements of fresh air, light, and isolation which we seem pledged not to lower or relax. England, it is true, has been for a full generation pedantic in the matter of what is called sanitary science. But England has every right to be. It is England's pedantry in this department of hygiene which has saved from certain dangers whole tracts of Europe. It is the example of the hotel with "English sanitation" which in Paris, Switzerland, the Riviera and elsewhere, has raised the level of the health-precaution standard throughout the Continent. It is quite true that when the laws of our Hygienic Renaissance of fifty years ago were once established we became rather inclined to regard them as having been brought by some Moses from Heaven itself, and our temptation has been to accept them entire without permitting flexibility of judgment. But on the whole our faith, if sometimes blind, has been always courageous. Our cautiousness is sound, and, if we are to remain leaders of the world in the sphere of sanitation, we must not fear the charge of being old-fashioned. After all, the laws of Nature are old-fashioned, too.

### THE ROYAL INSTITUTE OF PUBLIC HEALTH.

The Royal Institute of Public Health will hold a Congress in Plymouth from May 31 to June 5, 1922, under the presidency of Earl Fortescue, Lord Lieutenant of Devon. In addition to conferences on various matters of importance to Public Authorities, the Congress will be conducted in four sections, namely: Section I., "State Medicine and Municipal Hygiene"; President, D. Llewelyn-Williams, M.C., F.R.C.S., D.P.H., Medical Member of the Welsh Board of Health. Section II., "Naval, Military and Air"; President, Surgeon Rear-Admiral Sir Daniel McNabb, K.B.E., C.B., Principal Medical Officer of the Royal Naval Hospital, Plymouth. Section III., "Bacteriology and Bio-Chemistry"; President, Professor J. Martin Beattie, M.D., Professor of Bacteriology in the University of Liverpool. Section IV., "Women and Public Health"; President, Viscountess Astor, M.P. The Harben Lectures will be delivered during the meeting by Dr. T. Madsen, of Copenhagen.

In the Lecture Hall of the Royal Institute of Public Health the last four lectures of a course on "Tuberculosis and Public Health" will be given on Wednesdays, at 4 p.m. The subjects and lecturers are: March 1, "Surgical Tuberculosis in Children" (Mr. Charles E. M. Jones, M.B., M.R.C.S., L.R.C.P., Assistant Medical Officer, Lord Mayor Treloar Cripples' Hospital, Alton); March 8, "The Clinical Difference in the Course of Tuberculosis seen in Various Age Groups and Races" (Professor S. Lyle Cummins, C.B., C.M.G., M.D., Professor of Tuberculosis in the University College of S. Wales and Monmouth); March 15, "The Organisation and Conduct of Tuberculosis Dispensary Work" (Mr. W. H. Dickinson, O.B.E., M.B., D.P.H., Tuberculosis Medical Officer for the City and Council of Newcastle-on-Tyne); March 22, "The Helio-Alpine Treatment of Surgical Tuberculosis" (Mr. Andrew J. Morland, Assistant to Dr. Rollier at the Clinics for Surgical Tuberculosis, Leysin, Switzerland).

